# Strategies to improve γδTCRs engineered T-cell therapies for the treatment of solid malignancies

**DOI:** 10.3389/fimmu.2023.1159337

**Published:** 2023-06-27

**Authors:** A. D. Meringa, P. Hernández-López, A. Cleven, M. de Witte, T. Straetemans, J. Kuball, D. X. Beringer, Z. Sebestyen

**Affiliations:** ^1^ Center for Translational Immunology, University Medical Center Utrecht, Utrecht University, Utrecht, Netherlands; ^2^ Department of Hematology, University Medical Center Utrecht, Utrecht University, Utrecht, Netherlands

**Keywords:** T-cell therapy, γδTCR, cancer, immune therapy, antigen, fitness, TME (tumor microenvironment), migration

## Introduction

After the overwhelming clinical success of targeting hematological malignancies with CAR-T cells (1), the first signals of treatment are seen for solid tumors targeted by engineered immune cells (2). However, targeting solid tumors with this kind of immunotherapy still remains a challenge (3, 4). There are multiple mechanisms that make it difficult for adoptive cellular therapies to effectively target solid tumors.

First, most solid tumors lack homogeneous expression of a tumor-specific antigen making it difficult to find appropriate receptors to target them (5). The selection of targetable tumor antigens needs careful consideration to avoid targeting of healthy tissue, especially when considering engineered cellular therapies against solid cancers, where potent and safe antigens are rare (6). Additionally, the microenvironment of solid tumors holds unique features such as expression of immunosuppressive molecules and hypoxia that have a huge impact on T cell fitness (4, 7, 8). Finally, a combination of extracellular matrix deposition and anti-inflammatory signals, like attracting mesenchymal derived suppressor cells (MDSCs), prevent effective infiltration of T cells towards the tumor site (9).

In this article we will further discuss the roadblocks facing successful implementation of T cell therapies for the treatment of solid malignancies focusing on γδT cells and their receptors since they provide a new avenue to target novel tumor antigens. Characterization of these cells and their receptors holds the potential to generate novel strategies for targeting cancer and provide new engineering strategies to potentially overcome these hurdles.

## Gamma delta T cells as source of novel tumor-targeting receptors

The infiltration of γδT cell in tumors has been associated in many studies to have a favorable impact on patient survival (10–16), while some other studies made in murine models report that interleukin-17 (IL-17) producing γδT cells are tumor promoting (17, 18). While these data are very insightful, it has to be carefully handled when translating it to human clinical practices given that human and mouse γδT cell repertoires and functions are not fully compatible. Regardless of the ultimate effector function, activation of γδT cells is contingent upon the engagement of their surface receptors with antigens on the tumor cell. γδ T cells can be divided into two groups, Vδ2^+^ and Vδ2^-^, with Vδ1 forming the majority of Vδ2^-^ T-cells. Vδ2^-^ T cells are predominantly found in peripheral tissue and have also been shown to be enriched in carcinomas (11, 19–21). Multiple studies reported a correlating favorable clinical outcome either with the presence of Vδ2^-^ T-cells (12, 13) or with γδT in general (13). This tissue-association might be advantageous for targeting and infiltrating solid tumors when using Vδ1TCR T cells as effector cells. Vδ2^-^ TCRs can recognize a wide variety of ligands that are expressed on infected and malignant cells (22). A large number of studies have shown that numerous Vδ2^-^ TCRs can recognize nonpolymorphic MHC I-like molecules MR1 and CD1 (23, 24). Most CD1 isoforms, CD1a, CD1b, and CD1c, are mainly found on cells of hematological origin and declassify them as potential ligands for solid tumors (25), but both MR1 as CD1d have been found to be expressed on solid tumors (25, 26). Other γδTCR ligands expressed on solid tumors and are recognized by specific Vδ2^-^ TCR clones are endothelial protein C receptor (EPCR) (27), Annexin A2 (28), and EphA2 (29). Based on the wide breath of ligands recognized by Vδ2^-^ TCRs (22), it is to be expected that many more ligands for this subset will be identified in the future. While many of these Vδ2^-^ TCR ligands are also expressed on the surface healthy cells, such as EPCR on endothelial cells (30) and CD1d on APCs (31), no major safety concerns have been reported. For example, a study demonstrating that while an EPCR reactive Vδ2^-^ TCR clone recognized cytomegalovirus (CMV)-infected or malignant endothelial cells it was not reactive against normal endothelial cells, due to increased expression of immune modulating molecules such as CD54 and CD58 (27). Additionally, to avoid toxicity towards healthy, antigen presenting cells (APCs), lipid-specific CD1d reactive Vδ2^-^ TCRs can be used (32).

Unlike above discussed Vδ2^-^ T cells, Vδ2^+^ T cells, also referred as Vγ9Vδ2 T cells are mainly present in blood and their role of cancer immune surveillance have been studied the most among all γδT cells (33). The process of identifying the ligand complex for the invariant Vγ9Vδ2 TCRs has been a long and winding path, that started with the identification of phosphoantigens (34) that are bound by the intracellular domain of butrophylin 3A1 (BTN3A1) (35). This process leads to a re-localization of BTN3A1 to the cell surface (36, 37), where it can form a complex with BTN2A1 (38–40). Only when this phosphoantigen driven complex of BTN3A1 and BTN2A1 is formed on the plasma membrane, Vγ9Vδ2 TCRs can be activated. This multistep ligand complex formation serves a safety threshold that prevents Vγ9Vδ2 TCR mediated toxicity towards healthy tissue but enables the eradication of tumors in many preclinical models (41–43).

While γδT cells have their natural potential to target cancer, as described above, the most clinical trials to date, that have assessed the efficacy and safety of γδT cells as adoptive cellular therapy did show moderate clinical efficacy (44–47) where only incidentally e.g. prolonged survival of patients has been reported (46). However, the potential of natural, tumor infiltrating γδT cells has recently been demonstrated in colorectal cancer (10) and kidney cancer (16), supporting the idea to further investigate the details of receptors present on γδT cells for the treatment of cancer. While providing an emerging universe of tumor specific receptors, one has to carefully assess possible toxicity against healthy tissues in advanced 3-dimensional preclinical models (41, 42, 48) that resemble the homeostatic environment of the human body.

## Improving T-cell fitness for durable tumor control

T cell dysfunction has been one of the major causes of failure of CAR-T cell treatments as it results in poor T cell expansion and short-term persistence resulting in reduced anti-tumor efficacy (8, 49). Despite efforts to improve CAR designs, CAR-T cell exhaustion remains one of the main limitations of this kind of therapy (50–52). Thus, although CAR-T field has significantly growth in the last years, some studies advocate for the use of natural TCR signaling to reduce exhaustion of T cells (53, 54). The main reason for this is that CAR’s artificial design accelerates exhaustion of T cells when compared to TCR based therapies, mostly due to the described tonic signaling in the absence of antigen (54–56). In this line, several designs have been explored to make CAR more TCR-like, such as HLA-independent TCR (HIT) or synthetic TCR and antigen receptor (STAR) (57, 58). The CAR scFv sequence in these receptors is fused to the constant domains of an αβTCR, thereby preserving TCR signaling while using the CAR’s ability to recognize tumors in an HLA independent way. An elegant alternative to these designs is engineering αβ T cells to express tumor-reactive Vγ9Vδ2 TCRs (called TEGs) (41, 59). In this way, the use of γδTCRs in T cell therapy appear to be advantageous when compared with CARs or αβTCRs, as they supply T cells with natural TCR signaling while preserving the ability of recognize tumors in an HLA-independent way (44).

Optimal co-stimulation has been described as key to overcome exhaustion and improve T cell fitness and persistence in the context of cancer (60–62). Therefore, as costimulatory signals are highly involved in T cell metabolic reprogramming (63, 64) and T cell exhaustion is closely related with metabolic dysfunction, manipulation of co-stimulation in T cell therapies will result in improved metabolic T cell fitness, which is key to achieve robust anti-tumor responses (63). One example is the addition of co-stimulatory domains to the first generation of CARs, which has shown to improve persistence of these cells (65, 66). This led to the development of second and third generation of CARs with improved proliferation ability. Therefore, combining natural TCR signaling properties, by using γδTCRs to target tumors, with improved co-stimulation might be the answer to CAR-T limitations.

One way to improve the co-stimulation of T cells can be achieved by expressing chimeric costimulatory receptors (CCRs) in combination with a CAR or a TCR (67–70). These receptors preserve the structure of conventional second-generation CARs but lack the CD3ζ domain, therefore providing only costimulatory signals to the T cell. Uncoupling of signal 1 (CD3 signal) and signal 2 (co-stimulation) by this dual targeting has been shown to be beneficial (71–73) as T cells will only activate once synergistic signals are delivered upon encounter of both antigens. While these receptors improve T cell proliferation, they also reduce exhaustion (71) thereby improving T cell persistence in the tumor niche and leading to an improved therapeutic effect (71, 74).

A type of CCRs are the so-called switch chimeric co-receptors (75–78), which use the extracellular domain of a described inhibitory receptor (such as PD-1 or TIGIT) and link it to the intracellular domain of activating costimulatory receptors (such as CD28 or 4-1BB) or eventually DAP10, when expressed in γδT cells (70). Thus, these receptors turn inhibitory signals, that would normally induce exhaustion of T cells, into activating signals. This strategy improves not only T cell fitness, by improving co-stimulation, but also makes engineered T cells resistant to tumor microenvironment immunosuppressive factors.

Finally, it is important to further investigate the mechanisms that impact T cell fitness as not all the T cells subsets respond equal to the same stimulus. For example, TGF-β has been shown to improve cytotoxic activity of Vδ2^+^ T cells (79) while it is been described to suppress αβ T cells function (80). Furthermore, IL-15 has been shown to improve tumor killing capacity of γδT cells isolated from AML patients (81). Therefore, comprehensive studies and rational engineering it is key to develop effective therapies. In conclusion, to achieve durable anti-tumor responses the next generation of T cell-based immunotherapies should include fine-tuning of co-stimulation, to preserve T cell fitness, ensure persistence, and skew the T cells to the most potent phenotype.

## Tackling the tumor microenvironment

The lack of efficacy observed for different T cell treatments targeting various antigens in solid tumors suggest the presence of general barriers that inhibit the efficacy of these immunotherapies. The cellular and extra-cellular composition of the tumor microenvironment can influence the tumor biology and response to immune therapy (82). The dense extracellular matrix (ECM) of solid tumors is a physical barrier for T cells to penetrate leading to low numbers of infiltrating, endogenous T cells in solid tumors (4). Meanwhile, immunosuppressive cells such as myeloid-derived suppressive cells and regulatory T cells in the tumor microenvironment (TME) inhibit antitumor activity of T cells that do infiltrate in the TME (83). Different engineering strategies are being developed to overcome these general barriers of T-cell therapies in solid malignancies.

Modulation of the chemokine signaling of the tumor-reactive T cells can lead to improved T cell infiltration by increasing chemotaxis towards the tumor site. For example, expression of the colony stimulating factor receptor (CSF-R) in CAR-T cells improved migration towards solid tumor models producing CSF (84). Arming T cells with other chemokine receptors have shown similar results where CCR4, CCR2b and CXCR3 overexpression in the T cell products led to increased infiltration in the TME and thereby increased tumor targeting (85–87).

Upon infiltration of immune cells in the TME, multiple mechanisms can render the T cells inactive via expression of immunosuppressive molecules. Well-known checkpoint molecules such as PD-1 and TIM3 are not only affecting αβ T cells but also act on γδ T cells as has been recently shown (10) in colorectal cancer. However, γδ T cells are also often regulated by unique sets of inhibitory natural killer (NK) receptors: for example, tumor and stromal cells can express ligands for immune checkpoints in T cells like HLA-E binding NKG2A on γδ tumor infiltrating lymphocytes (TILs) (88). To overcome this, numerous cytokines have been tested to make armed CAR-T cells also known as T cell redirected for antigen-unrestricted cytokine-initiated killing (TRUCKs) (89). CAR-T cells targeting different solid tumor models were shown to improve their anti-tumor activity, increase their resistance to regulatory T cell signaling and improve local proliferation upon arming the T cells with IL-12 expression (90–92). Expression of other cytokines such as IL-7, IL-15 and IL-18 have shown to provide similar results by increasing therapy efficacy via increasing local inflammation in the TME (92–94). Chemokine and cytokine arming of γδTCR based T cell therapies could increase efficacy since other T cell engineering approaches for CAR-T cells.

Additionally, CAR-T cells can be engineered to express ECM-modifying enzymes to facilitate better penetration to the tumor site. Heparinase expressing GD2 CAR-T cells improved their infiltrating capacity in solid tumor models compared to CAR-T cells lacking heparin expression (95, 96). Arming CAR-T cell with prolyl endopeptidase is another approach for targeting the ECM in the TME (97). Expression of prolyl endopeptidase in CAR-T cells improved their anti-tumor activity, however some toxicity towards healthy tissue was observed with both ECM targeting approaches. Introducing these types of modifications could be very promising for improving the therapeutic effect of γδTCR T cells in solid tumors.

## Future perspectives

**Figure 1:**
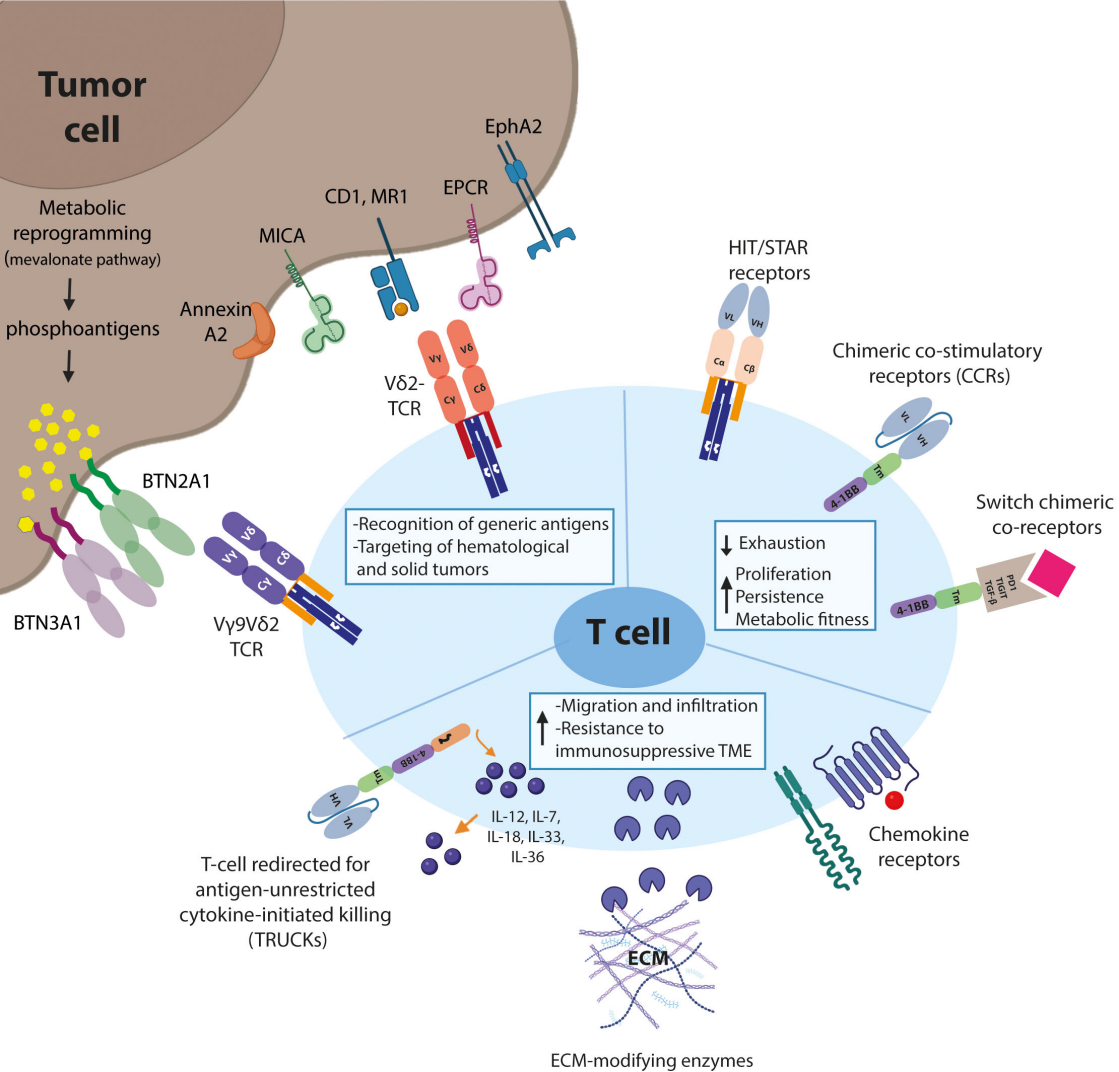
Schematic representation of T-cell engineering approaches. Biological mechanisms that prevent effective adoption of gd T-cell therapies for the treatment of solid malignancies and suggested engineering strategies to overcome these hurdles are shown.

Current developments in the field of engineered adoptive cellular therapies, especially CAR-T cell therapies show promising results in the treatment of haematological malignancies; more specifically B cell-derived tumors. However, adapting these T cells therapies to solid tumor treatments options requires overcoming certain impediments posed by solid malignancies and their TME (**Figure 1**). Fortunately, these T cells-based therapies allow for *ex vivo* modifications of the treatment to address these tumor-specific challenges posed in the TME of solid tumors where lesson learned from tumor specific γδT may provide a possible solution.

Selection of the tumor-reactive receptor and the tumor specific/associated antigen remains the first important step in optimizing T cell therapies in solid tumors. To this end, γδTCRs are an interesting option due to their unique recognition patterns. Secondly, the addition of a co-stimulatory signal, especially in combination with a naturally low affinity γδTCR can help improve T cell fitness via either one of the three suggested signalling approaches. Expressing a chimeric costimulatory receptor to mimic signal 2 will help the T cells to retain their anti-tumor activity upon prolonged exposure in the TME. Furthermore, the induction of inflammation via secretion of cytokines such as at the tumor site can help the tumor infiltrating γδTCR T cells to overcome the immunosuppressive signals present in the TME. Finally, expression of chemokine(receptors) or ECM modifying molecules can help increase T cell infiltration in the solid tumor microenvironment.

In conclusion, promising approaches for improving the efficacy and scope of T cell therapies are being developed to overcome the current roadblocks in the treatment of solid malignancies. Using γδTCRs as tumor-reactive receptors, and combining these with appropriate co-stimulation via expression of additional chimeric costimulatory receptor to improve fitness and providing additional mechanisms to improve γδTCR T-cell infiltration like boosting chemotaxis, will be key assets to enhance efficacy of T cell therapies for solid malignancies. While further modifying the T cells does contain risks, these solutions will help to optimize efficacy of engineered T cell therapies and introduce this technology for a more widespread use in anticancer therapy.

## Author contributions

All authors listed have made a substantial, direct, and intellectual contribution to the work and approved it for publication.
